# My first time: initiation into injecting drug use in Manipur and Nagaland, north-east India

**DOI:** 10.1186/1477-7517-4-19

**Published:** 2007-12-05

**Authors:** Michelle Kermode, Verity Longleng, Bangkim Chingsubam Singh, Jane Hocking, Biangtung Langkham, Nick Crofts

**Affiliations:** 1 Nossal Institute for Global Health, University of Melbourne, Carlton, Victoria, Australia; 2 c/- Project ORCHID, CBCNEI Mission Compound, Guwahati, Assam, India; 3 Key Centre for Women's Health, University of Melbourne, Carlton, Victoria, Australia

## Abstract

**Background:**

The north-east Indian states of Manipur and Nagaland are two of the six high HIV prevalence states in the country, and the main route of HIV transmission is injecting drug use. Understanding the pathways to injecting drug use can facilitate early intervention with HIV prevention programs. While several studies of initiation into injecting drug use have been conducted in developed countries, little is known about the situation in developing country settings. The aim of this study was to increase understanding of the contextual factors associated with initiation into injecting drug use in north-east India, and the influence of these factors on subsequent initiation of others.

**Method:**

In mid 2006 a cross-sectional survey among 200 injecting drug users (IDUs) was undertaken in partnership with local NGOs that provide HIV prevention and care services and advocacy for IDUs in Imphal, Manipur and Dimapur, Nagaland. The questionnaire elicited detailed information about the circumstances of the first injection and the contexts of participants' lives. Demographic information, self-reported HIV status, and details about initiation of others were also recorded.

**Results:**

Initiation into injecting drug use occurred at 20 years of age. The drugs most commonly injected were Spasmo-proxyvon (65.5%) and heroin (30.5%). In 53.5% cases, a needle belonging to someone else was used. Two-thirds (66.7%) had used the drug previously, and 91.0% had known other IDUs prior to initiation (mean = 7.5 others). The first injection was usually administered by another person (94.5%), mostly a friend (84.1%). Initiation is a social event; 98% had others present (mean = 2.7 others). Almost 70% of participants had initiated at least one other (mean = 5 others). Initiation of others was independently associated with being male and unemployed; having IDU friends and using alcohol around the time of initiation; and having been taught to inject and not paid for the drug at the time of initiation.

**Conclusion:**

Targeting harm reduction messages to (non-injecting) drug users and capitalising on existing IDU social networks to promote safe injecting and deter initiation of others are possible strategies for reducing the impact of injecting drug use and the HIV epidemic in north-east India.

## Introduction

The north-east Indian states of Manipur and Nagaland, which lie along the border with Myanmar, are characterised by ethnic conflict, armed civil insurgency, a heavy military presence and high unemployment [1]. Classified by the Indian National AIDS Control Organisation (NACO) as high HIV prevalence states, they make up 0.4% of India's population, but account for 3.0% of cumulative AIDS cases [2]. Injecting drug use is a serious public health problem in both states, where heroin and Spasmo-Proxyvon (a synthetic opioid analgesic) are the most commonly injected drugs [3]. Injecting drug use is a major route of HIV transmission in this region.

Although Manipur and Nagaland are neighbouring north-east Indian states, they are different from each other in a number of important ways including ethnicity, culture, religion, insurgent movements, patterns of drug use and HIV, and the extent to which harm reduction approaches are accepted and integrated into the local response. Both the HIV epidemic and the public health response to it are more mature in Manipur. Sentinel surveillance data estimate that HIV prevalence among injecting drug users (IDUs) in Manipur was 24% in 2005, but only 4.5% in Nagaland [4]. However, HIV infection is not limited to people engaging in risk behaviours in either state; more than 25% of districts in these two states report > 1% HIV prevalence among antenatal attendees [3].

Approximately 2% of the population in Manipur and Nagaland engage in injecting drug use [3]. The vast majority are male, and the socio demographic profile differs substantially from that in other parts of India; IDUs in the north-east are more likely to be well-educated, younger, and reman living with their families [5]. The proportion who are female is estimated to be around 7% [6]. Almost half of IDUs in Manipur (47%) are initiated into injecting before the age of 21 years (compared to 24% for India), and 86% by 26 years (compared to 56% for India) [5].

Current harm reduction programs in Manipur and Nagaland aim to reduce the risk of HIV infection among IDUs by offering a range of services including needle and syringe distribution and condom promotion. However, these programs are constrained in what they are able to achieve as both states are characterised by deeply felt social conservatism. There is an absence of interventions to address upstream factors that contribute to young people's decision to inject drugs. Transitioning from non-injecting drug use to injecting is not inevitable, and a range of individual and social network factors have been shown to influence this in developed country settings [7-16]. Factors associated with commencing to inject drugs include: homelessness [7,9]; unemployment [7]; younger age at first heroin use [7,9]; having a sexual partner who has injected [7,14]; social network influences [9]; physical abuse [9]; and perceived endorsement of friends [7,9]. Subjective reasons for starting to inject include: curiosity [8,13,15]; pleasure seeking [12]; wanting the (better) high [8,13,15]; peer pressure [13,15]; economic [8,12,13]; and knowing other injectors [8].

Two Australian studies of initiation into injecting drug use investigated factors associated with the initiation of others [10,11]. They found that 37% and 47% of their respective samples had gone on to initiate others, and that initiators had been injecting for longer, and were more likely to share injecting equipment, inject multiple drugs, deal drugs, and be unemployed.

In contrast, little is known about initiation into injecting drug use in developing country settings where the legal, political, socio-cultural and economic contexts are very different, as are the epidemiology of HIV and the patterning of injecting drug use. A cross-sectional study in Thailand among 2231 drug users found that being ≥ 20 years, being single, having received education, living in an urban area, having a history of smoking or incarceration, having multiple sexual partners, having experienced sexual abuse, using heroin (rather than amphetamines), and younger age of drug initiation were significantly associated with transition to injecting [17].

The aim of this study was to increase understanding of the contextual factors associated with initiation into injecting drug use in Nagaland and Manipur, and the influence of these factors on subsequent initiation of others into injecting. Understanding the pathways to injecting drug use will assist with identification of groups at risk of initiation into injecting, as well as mechanisms of initiation, so that prevention interventions can be better designed and targeted. Importantly, the findings from this study will also highlight opportunities for providing harm reduction interventions earlier in the 'career' of IDUs, and can be used to inform and enhance social and political advocacy for HIV prevention.

## Methods

This cross-sectional survey was conducted among IDUs from Imphal, Manipur and Dimapur, Nagaland between May and July 2006. The survey was conducted in collaboration with three Indian non-governmental organisations (NGOs): Social Awareness Service Organisation (Manipur); Bethesda Youth Welfare Centre (Nagaland); and Community Awareness Development Foundation (Nagaland). These NGOs provide a range of services for IDUs including needle and syringe programs, peer education, primary health care, counselling and rehabilitation. Qualitative data were also collected via in-depth interviews, and findings from that component of the study will be reported elsewhere.

### Sampling

A total of 200 IDUs aged ≥ 18 years were surveyed. An IDU was defined as a person who has injected illicit drugs within the last three years. The sampling approach used a combination of convenience and snowball sampling and was stratified by state (half from each state) and sex (10% were female). In an Australian study of initiation into injecting drug use, 47% of IDUs had initiated at least one other person into injecting [10]. We assumed that 50% of IDUs in north-east India had initiated at least one other person, and for the 95% confidence interval to be 50% ± 7 percentage points, the sample size required was 195.

### Questionnaire development

The questionnaire was based on one used previously to describe initiation into injecting in Melbourne, Australia [10], but was adapted for the local context and piloted. It was interviewer administered and the questions covered a range of topics: demographic information; detailed information regarding the circumstances of the first injection of illicit drugs – both what happened at the time of the injection specifically and what was happening in the person's life more generally; previous and current drug use; self-reported HIV and hepatitis C status; and initiation of others into injecting drug use. The questions were in English but were translated into the local language as required. The appropriate phrasing of each question in the local language was thoroughly discussed with the bi-lingual interviewers during the training and piloting phases.

### Data collection

Eleven bi-lingual peer outreach workers (ORWs) were trained to collect the data. The ORWs were supervised and supported by a locally appointed research officer. Individual IDU clients were approached by the ORWs in the course of their work. Although not formally recorded, feedback from the ORWs indicated that the number of IDUs refusing to participate was very small. Participants were paid Rs100 (~USD 2.3) for their time. Interviews took place in a range of settings including drop-in-centres, clients' homes, hotels and tea-shops.

### Data analysis

The data were entered into Microsoft Excel 2003 and analysed using Statistical Package for the Social Sciences (SPSS) Version 14.0. The statistical tests used to assess the strength of associations between variables included Pearson's chi-square and t-test for independent samples. Nagaland and Manipur have different histories, ethnicities and religion, so all variables were analysed for state differences. Variables associated with being an IDU who initiates others were investigated using logistic regression, and odds ratios and 95% confidence intervals (CIs) were calculated. A stepwise technique was used, and variables were selected for inclusion in the model on the basis of their p value. A model of best fit was chosen with variables included on the basis of a change in the log likelihood at p < 0.1.

### Ethical issues

The study was funded by the United Kingdom's Department for International Development (DFID) through the Research and Learning Fund. It was approved by the Human Research Ethics Committee at the University of Melbourne, Australia, and the Institutional Review Board of the Emmanuel Hospital Association, New Delhi, India. All participants gave verbal informed consent and confidentiality was assured. No participant names were recorded at any stage of the study.

## Results

### Demographic information and current life situation

The mean age of participants was 24.5 years (range 19–28, SD 2.17). The majority identified ethnically as Naga (45.5%) or Meitei (39.5%). More than half were Christian (57.0%) and 40.0% were Hindu (Table 1). Due to oversight the question about marital status was only asked in Nagaland where 66% were single and 34% were or had been married. Slightly more than half had either not attended or not finished school (52.0%), and 11.0% had graduate or post-graduate qualifications (Table 1).

**Table 1:** Demographic information by state

**Variable**	**Total (n = 200)**		**Manipur (n = 100)**		**Nagaland (n = 100)**		**p value**
	**%**	**n**	**%**	**n**	**%**	**n**	
**Ethnicity**							
- Meitei	39.5	79	79.0	79	0	0	< 0.01
- Naga	45.5	91	4.0	4	87.0	87	
- Kuki	6.0	12	12.0	12	0	0	
- Other	9.0	18	5.0	5	13.0	13	
**Religion**							
- Hindu	40.0	80	74.0	74	6.0	6	< 0.01
- Muslim	1.0	2	0	0	2.0	2	
- Christian	57.0	114	22.0	22	92.0	92	
- Other	2.0	4	4.0	4	0	0	
**Education**							
- None	4.5	9	4.0	4	5.0	5	= 0.06
- Schooling not completed	47.5	95	38.0	38	57.0	57	
- Schooling completed	37.0	74	46.0	46	28.0	28	
- Graduate	9.5	19	11.0	11	8.0	8	
- Post-graduate	1.5	3	1.0	1	2.0	2	
**Employment**							
- Employed	17.9	35	11.5	11	24.0	24	= 0.02
- Unemployed	82.1	161	88.5	85	76.0	76	
Other sources of income*							
- Family	83.8	165	93.0	93	74.2	72	< 0.01
- Partner	9.1	18	12.0	12	6.2	6	= 0.16
- Friends	44.7	88	71.0	71	17.5	17	< 0.01
- Selling drugs	32.0	63	48.0	48	15.5	15	< 0.01
- Stealing	38.1	75	56.0	56	19.6	19	< 0.01
- Pawning goods	54.8	108	78.0	78	30.9	30	< 0.01
- Sex work	6.1	12	4.0	4	8.2	8	= 0.21
- Other†	16.8	33	17.0	17	16.5	16	= 0.92
**Housing**							
- Homeless	2.0	4	0	0	4.0	4	< 0.01
- Living with family	70.5	141	86.0	86	55.0	55	
- Living with friend	0.5	1	0	0	1.0	1	
- Renting	18.5	37	12.0	12	25.0	25	
- Other	8.5	17	2.0	2	15.0	15	

*More than one response possible

†E.g. driving/pedalling a rickshaw, casual labouring, small business (eg. vegetable selling) and gambling

Only 17.9% of participants were employed. The mean monthly income was Rs3662 (~USD 84) (range 200–20000, median 3000, SD 31.3). Participants were asked the source of their income other than through employment and most received money from their family (83.8%). State differences in all of these variables were observed (Table 1).

The majority of participants (70.5%) lived in their family home and 72.9% were sharing their living space with their parents. Only 14.6% lived with a partner/wife/husband, 12.6% with other relatives, 6.5% with friends, 4% with others such as parents-in-law, and 1.5% lived alone (multiple responses allowed). One quarter (25.1%) had children of their own. The average number of children was 1.5 (range 1–3, SD 0.7), and 78.6% of those with children were currently living with them. In terms of mobility, all participants were born in north-east India, but 38% were currently residing in a district that was not their birthplace.

### Description of initiation into injecting drug use

The mean age of the first injection of illicit drugs was 20.1 years (range 13–26, median 19, SD 2.4). There was no difference in age of first injection by state or by drug injected. For their first injection, 65.5% of participants injected SP and 30.5% injected heroin. Two-thirds (66.7%) had used the drug previously. In 53.5% of cases the needle used for the first injection belonged to someone else (Table 2).

**Table 2:** Circumstances of first injection of illicit drugs by state

**Variable**	**Total (n = 200)**		**Manipur (n = 100)**		**Nagaland (n = 100)**		**p value**
	**%**	**n**	**%**	**n**	**%**	**n**	
**Drug injected 1^st ^time**							
- Heroin	30.5	61	48.0	48	13.0	13	< 0.01
- SP	65.5	131	49.0	49	82.0	82	
- Other*	4.0	8	3.0	3	5.0	5	
**Had used this drug previously**							
- No	33.3	66	40.0	40	26.5	26	= 0.04
- Yes	66.7	132	60.0	60	73.5	72	
**Whose needle was used**							
- Mine	44.5	89	52.0	52	37.0	37	= 0.04
- Someone else's	53.5	107	45.0	45	62.0	62	
- Don't know	2.0	4	3.0	3	1.0	1	
**Whose idea to inject**							
- Mine	32.5	65	32.0	32	33.0	33	= 0.77
- Someone else's	58.0	116	57.0	57	59.0	59	
- Both	9.5	19	11.0	11	8.0	8	
**Planned or spontaneous**							
- Planned	21.0	42	11.0	11	31.0	31	= 0.01
- Spontaneous	79.0	158	89.0	89	69.0	69	
**Who paid for the drug**							
- Me	48.5	96	40.8	40	56.0	56	= 0.01
- Someone else	32.3	64	33.7	33	31.0	31	
- Both	13.1	26	14.3	14	12.0	12	
- Free	6.1	12	11.2	11	1.0	1	
**Who obtained the drug**							
- Me	23.1	46	14.1	14	32.0	32	< 0.01
- Someone else	63.3	126	72.7	72	54.0	54	
- Both	13.6	27	13.1	13	14.0	14	
**Place of 1^st ^injection**							
- My house	17.5	35	15.0	15	20.0	20	= 0.09
- Friend's house	43.5	87	50.0	50	37.0	37	
- Peddler's place	2.5	5	2.0	2	3.0	3	
- Public toilets	3.5	7	6.0	6	1.0	1	
- Hotel/restaurant	1.5	3	1.0	1	2.0	2	
- Bushes/wasteland	15.5	31	9.0	9	22.0	22	
- Riverbank	3.5	7	4.0	4	3.0	3	
- Other†	12.5	25	13.0	13	12.0	12	
**Concurrent use of other drugs**							
- No	46.7	93	50.0	50	43.4	43	= 0.35
- Yes	53.3	106	50.0	50	56.6	56	
**Drugs used concurrently‡**							
- Alcohol	10.6	21	8.0	8	13.1	13	= 0.24
- Marijuana	6.5	13	11.0	11	2.0	2	= 0.01
- Heroin (smoked)	7.0	14	5.0	5	9.1	9	= 0.26
- SP (oral)	21.6	43	20.0	20	23.2	23	= 0.58
- Cigarettes	37.2	74	43.0	43	31.5	31	= 0.09
- Glue	3.0	6	5.0	5	1.0	1	= 0.10
- Gutka (packaged paan)	21.6	43	19.0	19	24.2	24	= 0.37
- Pills	7.0	14	5.0	5	9.1	9	= 0.26
- Other	5.0	10	7.0	7	3.0	3	= 0.20
**Others present**							
- No	2.0	4	1.0	1	3.0	3	= 0.31
- Yes	98.0	196	99.0	99	97.0	97	
**Injected by other**							
- No	5.5	11	3.0	3	8.0	8	= 0.12
- Yes	94.5	189	97.0	97	92.0	92	

*E.g. pentazocine, morphine

†E.g. hostel, college, jail, stadium, cemetery and plantation

‡More than one response possible

Participants were asked whose idea it was to inject, whether or not the event was planned or spontaneous, who paid for the drug, who obtained it and where the first injection took place. The idea to inject was most often someone else's (58.0%). For the majority (79.0%), the first injection was remembered as happening spontaneously. Almost half of the participants paid for the drug themselves (48.5%) or shared the cost with another (13.1%). The drug was most often obtained by someone other than the participant (63.3%). The most common places for the first injection were a friend's house (43.5%) or the participant's own house (17.5%). Only 2.5% were initiated at the drug dealer's place. More than half of the participants (53.3%) had other drugs in their system at the time of the first injection (Table 2).

Initiation into injecting drug use was a companionable event. The mean number of other people present (not counting the participant) was 2.7 (range 0 – 25, median 2, SD 2.2), and most of those present also injected. Only 2.0% of participants were alone when they injected for the first time and 95.0% had up to five other people present. More than four-fifths (85.5%) had at least one friend present, 14% had at least one relative, 13.0% had at least one acquaintance/stranger, 3.5% had a drug dealer (known as 'peddlers' in north-east India), and 1.5% had a partner/wife/husband present the first time they injected (multiple responses allowed).

Most participants (94.5%) were injected by another person the first time, most commonly a friend (84.1%), followed by a relative (9%), an acquaintance/stranger (4.7%), a partner/wife/husband (1.6%) or a drug dealer (0.5%). They had known this person for an average of 7.0 years (range 2 hours – 21 years, SD 5.8 years). A very small proportion (2.9%) had known the person who initiated them for one month or less; 14.9% had known the person for between one month and one year; 18.3% had known them for between one and three years; and close to two-thirds (64.1%) had known the person who initiated them for more than three years. In 87.3% of cases the participant asked the other person to inject them. Another person prepared the drug 93.3% of the time, taught the participant how to inject 69.8% of the time, and told the participant about the need to use new injecting equipment 36.5% of the time.

All eleven participants who injected themselves the first time were shown how to inject by either friends or relatives. The participants had known this person for an average of 6.4 years (range 6 months – 20 years). In six cases the participant asked the other person to show them how to inject, in eight cases the participant prepared the drug, having been taught by another person in six of those cases, and in three cases the other person mentioned the need to use new injecting equipment.

A number of state differences in the circumstances of first injection were observed (Table 2). Those from Nagaland were more likely to have: injected SP; used the drug previously; used someone else's needle; planned the injection; and paid for and obtained the drug themselves.

### Reasons for injecting

Participants were asked an open-ended question about their reasons for injecting at that point in time. The reasons were coded as: curiosity about the high/pleasure seeking (46.7%); the influence of others (34.7%); economic reasons (10.1%); and other reasons (8.5%), which were mainly a reaction to a negative situation in their life, or not knowing that the drug could be used in any other way. The influence of others was expressed in two different ways. Firstly, there was a desire on the part of some participants to be the same as their friends who injected, and secondly some participants were urged by their friends to try injecting. The economic imperative to inject was due to a shortage of money or drugs and therefore a need to use the available drug more efficiently by injecting rather than chasing/smoking or taking it orally.

### Life situation at the time of initiation

Participants were asked a series of questions about their life situation at the time of initiation into injecting drug use. One-third of participants (35.5%) were either unemployed or had dropped out of school (Table 3). Most participants were living with their parents (80.0%) or other relatives (13%) at that time (Table 3).

**Table 3:** Life situation at the time of initiation into injecting drug use by state

**Variable**	**Total (n = 200)**		**Manipur (n = 100)**		**Nagaland (n = 100)**		**p value**
	**%**	**n**	**%**	**n**	**%**	**n**	
**School/employment status**							
- School student	34.0	67	51.5	51	16.3	16	< 0.01
- College student	18.8	37	22.2	22	15.3	15	
- Dropped out	29.9	59	19.2	19	40.8	40	
- Unemployed	11.7	23	5.1	5	18.4	18	
- Employed	5.6	11	2.0	2	9.2	9	
Living with*							
- Parents	80.0	160	84.0	84	76.0	76	= 0.16
- Parents-in-law	0.5	1	1.0	1	0	0	= 0.32
- Partner	4.0	8	3.0	3	5.0	5	= 0.47
- Relatives	13.0	26	14.0	14	12.0	12	= 0.67
- Friends	10.0	20	14.0	14	6.0	6	= 0.06
- Alone	1.5	3	1.0	1	2.0	2	= 0.56
- Other†	3.0	6	0	0	6.0	6	= 0.03
**Knew other IDUs**							
- No	9.0	18	5.0	5	13.0	13	= 0.05
- Yes	91.0	182	95.0	95	87.0	87	
**Previous trouble with police**							
- No	76.8	152	78.8	78	74.7	74	= 0.50
- Yes	23.2	46	21.2	21	25.3	25	
**Previous incarceration**							
- No	88.8	175	96.9	95	80.8	80	< 0.01
- Yes	11.2	22	3.1	3	19.2	19	

*More than one response possible

†E.g. hostels, jail, homeless

Almost all participants (91.0%) knew other IDUs prior to their initiation into injecting (Table 3). The mean number of IDUs known was 7.5 (range 0 – 50, SD 6.5). About one-tenth (9.2%) knew no other IDUs at the time of initiation; 74.9% knew between 1–10 IDUs; 13.8% knew 11–20 IDUs; and 2.0% knew more than 20 IDUs. The majority (83.3%) had at least one injecting friend, 55.7% knew of people in their neighbourhood who injected, 20.3% knew of relatives (other than immediate family), 16.7% had a brother who injected and 1.0% a partner.

Almost one-quarter of participants (23.2%) had previously been in trouble with the police prior to their first injection, on an average of two occasions (range 1 – 7, SD 1.5). Of those who had been in trouble with the police, 48.9% had been in prison (11.0% of the entire sample); on average 1.4 times (range 1–6, SD 1.1).

State differences in life situation at the time of initiation into injecting drug use were noted (Table 3). Participants from Nagaland were more likely to be out of school or unemployed and to have experienced a period of incarceration.

### Drug use history

Around the time of initiation into injecting drug use (as opposed to on the occasion of initiation outlined above) and during the last six months and the last week prior to the survey, use of multiple other drugs was commonplace (Table 4).

**Table 4:** Drug use history by state

**Variable**	**Total (n = 200)**		**Manipur (n = 100)**		**Nagaland (n = 100)**		**p value**
	**%**	**n**	**%**	**n**	**%**	**n**	
Drug use around the time of initiation into injecting drug use*							
Alcohol	80.0	160	85.0	85	75.0	75	= 0.08
Marijuana	50.5	101	72.0	72	29.0	29	< 0.01
Heroin†	43.0	86	58.0	58	28.0	28	< 0.01
SP†	65.5	131	68.0	68	63.0	63	= 0.46
Cigarettes	86.5	173	95.0	95	78.0	78	< 0.01
Glue	22.0	44	34.0	34	10.0	10	< 0.01
Gutka (packaged paan)	62.5	125	63.0	63	62.0	62	= 0.88
Pills	50.0	100	67.0	67	33.0	33	< 0.01
Other‡	14.0	28	16.0	16	12.0	12	= 0.41
Drug use in the last six months*							
Alcohol	59.5	119	67.0	67	52.0	52	= 0.03
Marijuana	38.0	76	59.0	59	17.0	17	< 0.01
Heroin†	57.0	114	72.0	72	42.0	42	< 0.01
SP†	82.0	164	75.0	75	89.0	89	= 0.01
Cigarettes	81.5	163	93.0	93	70.0	70	< 0.01
Glue	16.0	32	26.0	26	6.0	6	< 0.01
Gutka (packaged paan)	58.5	117	66.0	66	51.0	51	= 0.03
Pills	52.5	105	64.0	64	41.0	41	= 0.01
Other‡	14.5	29	24.0	24	5.0	5	< 0.01
Drug use in the last week*							
Alcohol	66.0	33	35.0	35	31.3	31	= 0.58
Marijuana	22.0	44	38.0	38	6.1	6	< 0.01
Heroin†	44.5	89	61.0	61	28.3	28	< 0.01
SP†	65.0	130	59.0	59	71.7	71	= 0.06
Cigarettes	68.0	136	82.0	82	54.5	54	< 0.01
Glue	8.0	16	14.0	14	2.0	2	< 0.01
Gutka (packaged paan)	48.0	96	55.0	55	41.4	44	= 0.06
Pills	29.5	59	28.0	28	31.3	31	= 0.61
Other‡	9.0	18	17.0	17	1.0	1	< 0.01

*More than one response possible

†Inclusive of all routes of administration

‡E.g. cough syrup, chewing tobacco, opium

Information about injecting drug use during the last six months and the last week was collected. During the preceding six months 46.7% had injected only SP, 18.1% had injected only heroin, 23.1% had injected both, and 12.1% had injected neither. As some survey participants were recruited from drug substitution programs, their injecting drug use during the last week was atypical. Excluding the 56 (28.3%) participants who had not injected any drug in the last week, 67.6% injected SP and 43.0% injected heroin. Of those participants who had injected in the last week, the average number of injections was 16.9 (range 1 – 70, SD 10.66). They spent an average of Rs149 per day (~USD 3.4) on injecting drugs (range 10 – 1000, median 95, SD 132.4).

Participants were asked about injecting risk behaviours. The majority (81.2%) said they rarely or never used a needle used by someone else, but 12.5% did use a needle used by someone else at least weekly. Among those who have on occasions used a needle or syringe used by someone else, 77.7% always cleaned it before use, most commonly with water and/or saliva.

A number of state differences in drug use history were observed (Table 4). Participants from Nagaland were consistently less likely than those from Manipur to report use of most drugs including alcohol, marijuana, heroin and cigarettes, but they were more likely to report use of SP.

### Knowledge of blood-borne pathogen transmission prior to initiation

Prior to initiation into injecting, almost three-quarters of participants (72.4%) had heard of HIV and AIDS, 27.0% had heard of hepatitis B and 18.6% hepatitis C, but only 67.5% knew that diseases could be spread by unsafe injecting practices. The majority (87.5%) said they knew where to obtain new needles and syringes at that time. Most participants (68.5%) said that they were not worried about HIV infection the first time they injected. The most common reasons for not being worried were: lack of knowledge about HIV (41.6%); a new needle/syringe was used (37.2%); the focus was exclusively on obtaining the drug and getting high (9.5%); feeling confident that their friend was not HIV infected (5.1%); and the injecting equipment was cleaned prior to use (2.9%).

### Participation in HIV and HCV testing

Overall, 48.7% reported having been tested for HIV and only 5.2% for HCV infection. Of the 91 participants who had been HIV tested, 79 shared the result with the interviewer, and 19.0% of these were positive. Of the ten participants who had been tested for HCV, four were negative, three were positive, and three chose not to share this information. Only 10.0% of participants reported that they were vaccinated against hepatitis B.

### Initiation of others

The majority of participants (69.3%) had helped someone else inject for the first time. This was unrelated to state, age, drug initially injected or length of time since first injection, but was associated with sex. Males were more likely to initiate others than females (73.5% *cf *35.0%, p < 0.01). The average number of people initiated was 5.0 (range 1 – 20, median 3, SD 3.76). This means that 138 study participants went on to initiate 690 others into injecting drug use. Logistic regression modelling was used to identify variables independently associated with being an initiator of others (Table 5). Initiators were more likely to: be male and unemployed; have had IDU friends and been an alcohol user around the time of their own initiation; and to have been taught how to inject and not paid for the drug at the time of their own initiation.

**Table 5:** Odds ratios (unadjusted and adjusted) for variables that predict initiating others into injecting drug use

**Variable**		**% initiating others**	**Unadjusted OR (95%CI)**	**Adjusted OR (95%CI)**
**Sex**				
	Male	73.2		1.0
	Female	35.0	0.20 (0.07–0.52)	0.28 (0.08–1.02)
**Employed**				
	No	75.6		1.0
	Yes	45.7	0.27 (0.13–0.58)	0.35 (0.14–0.84)
**IDU friends at time of initiation**				
	No	40.6		1.0
	Yes	76.1	4.65 (2.10–10.30)	5.11 (1.98–13.22)
**Using alcohol around time of initiation**				
	No	47.5		1.0
	Yes	74.8	3.29 (1.61–6.73)	3.47 (1.47–8.19)
**Taught to inject at initiation**				
	No	58.1		1.0
	Yes	74.5	2.10 (1.12–3.97)	2.65 (1.20–5.89)
**Paid for drug at initiation**				
	No	79.4		1.0
	Yes	58.9	0.37 (0.20–0.70)	0.27 (0.12–0.57)

### Sex differences

Although the number of females in the study is too small to draw any meaningful conclusions about them, a number of sex differences were observed that are briefly described here. Compared with males, the females in this study were more likely to be married (90.0% *cf *27.8%, p < 0.01), have children (64.7% *cf *20.7%, p < 0.01), and to have either never attended or dropped out of schooling (80.0% *cf *48.9%, p < 0.01). They were less likely to be living with their parents (20.0% *cf *78.8%, p < 0.01), had a higher monthly income (p < 0.01), and were more likely to be obtaining money from partners (35.0% *cf *6.2%, p < 0.01) and sex work (60.0% *cf *0.0%, p < 0.01). Consistent with anecdotal reports, females were more likely than males to have injected heroin the first time (60.0% *cf *27.2%, p < 0.01), and to have reported heroin use in the last six months (100.0% *cf *52.2%, p < 0.01), and the last week (80.0% *cf *40.8%, p < 0.01).

## Discussion

This study of initiation into injecting drug use in north-east India found that the first injection of illicit drugs was occurring around the age of 20 years, and the drugs most commonly injected were SP and heroin, although multiple other drugs were also being used. The first injection was usually administered by another person who was well-known to the person being initiated, generally a friend. This is similar to findings of other studies investigating initiation into injecting, where the majority were also injected by friends the first time [10,11,13,15].

Most of the IDUs in this study knew a range of other IDUs prior to initiation into injecting drug use including friends, neighbours or family members, many of whom had been known for a long time. It would seem that participation in injecting was somewhat normalised for these young people, and therefore perhaps not perceived as deviant behaviour, which may in turn make it easier for non-injecting drug users to progress to injecting. Initiation into injecting was overwhelmingly a social event as evidenced by the fact that almost all participants (98%) were with others at the time of initiation, and on average there was between three and four people present (including the person being initiated). The vast majority (85%) had at least one friend present. Other studies have identified social network factors as playing an important role in facilitating the transition to injecting [7,9,15]. Many participants took an active role in the process of initiation either by asking another person to inject them (87%), contributing to the cost of the drug (62%), participating in the decision to inject (42%), or actively planning the event (21%).

The main reasons for deciding to inject at that point in time were curiosity about the high, pleasure seeking, the influence of others, and needing to use the drug more economically. These reasons are similar to those identified in other studies of transitions from non-injecting to injecting drug use [8,10,12,13,15]. Socio-economic adversity is commonplace in north-east India, yet few participants identified escape from adversity as a factor influencing their decision to inject. However, the fact that this was not identified by participants does not mean that it is not an influential factor. It is possible that the contribution of socio-economic adversity to the problem of injecting drug use in north-east India is not perceived at the individual level.

Almost 70% of participants had initiated another person into injecting, which is a lot higher than the proportions in other studies (47% and 37%) [10,11]. It does appear that a sub-set of IDUs are more likely to initiate others, especially unemployed young men with social networks that include other injecting friends. Targeting this particular group with HIV prevention messages is very important as they are initiating a lot of other IDUs, and the time of initiation is clearly risky for blood-borne virus transmission, in part due to lack of knowledge about HIV/AIDS.

A number of limitations should be borne in mind when interpreting these results. The sample was not representative of IDUs from north-east India so the results should be generalised with caution. It may be the case that IDUs not in contact with NGOs and those from rural areas are different from the participants in this study in important ways that could be associated with initiation into injecting. Similarly, participants enrolled in oral substitution programs, who constituted up to one-quarter of the sample, may be different from other drug users. The small number of females in the study means that little can be said about them with confidence. More research is required to better understand the world of female IDUs in north-east India, especially given the recognised links between drug use and engagement in sex work. As the data were collected by peer workers from NGOs working in the area of harm reduction, social acceptability bias may have influenced some responses, especially those related to the safety of injections.

These findings have a number of public health implications. As the majority of participants had previously used the drug they initially injected, it may be appropriate to target harm reduction messages to (non-injecting) oral/inhalant drug users. This could include messages delivered by peers regarding disease transmission and safe injecting practices, as well as messages to deter progression to injecting. Social change among IDUs in relation to needle and syringe re-use has been achieved, at least to some extent, so other changes are potentially possible. The idea of using social networks among community sub-groups engaged in HIV risk behaviours in order to change social norms has been described by others as a possible strategy for harm reduction [18-21].

The proportion of participants who had participated in testing for blood-borne viruses was low, especially in the case of hepatitis C. While the availability of treatments is currently limited in this part of the world, the situation is gradually improving, and the more people that know they are infected with a hepatitis C, the more advocates for better access to treatment there will be, and the more others can be protected from infection.

A number of state differences were observed (see Tables 1, 2, 3 and 4) but as the study participants were not representatively sampled, it is difficult to know to what extent these are real state differences and to what extent they are a function of differing approaches to participant recruitment. However, some of the observed differences are consistent with anecdotal reports from those working in the field, and this strengthens the likelihood that at least some of these differences are real differences, highlighting the need for local knowledge and understanding in order to tailor HIV prevention programs to meet local needs. For example, the needle and syringe requirements of SP users are likely to be quite different from those of heroin users.

Looking further upstream, most people familiar with the socio-economic contexts of north-east India would agree that there is an urgent need to create challenging opportunities to meaningfully engage young men to deter them not only from injecting drug use but also from armed insurgency. Most of the male participants in this study were in their mid-twenties, and were single, unemployed and living with their parents who supported them financially. Even prior to initiation into injecting, 42% had dropped out of study or were unemployed. These findings create an image of groups of young men well connected to each other who have no adult responsibilities and a lot of free time in a context where there is a limited range of things to do, both recreationally and occupationally. It is probable that the vacuum created by this scenario is to some extent filled by drug use, and addressing this issue is a community-wide responsibility. Community-based interventions to reduce the level of drug use in developing country settings have been documented in China [22] and Thailand [23], although sustainability was found to be lacking in the latter. Further research to better understand initiation into (non-injecting) drug use and to identify factors that protect against progression to injecting is needed.

Current harm reduction interventions target relatively established IDUs and struggle for support from civil society in conservative contexts such as that found in north-east India. It may well be easier to mobilise civil society support for interventions that aim to prevent initiation into injecting drug use, and such interventions would complement and strengthen existing harm reduction programs.

## Competing interests

The author(s) declare that they have no competing interests.

## Authors' contributions

Michelle Kermode was primarily responsible for study conceptualisation and design, data collection, analysis and interpretation, and drafting and revising the manuscript. Verity Longleng and Chingsubam Bangkim Singh participated in data collection and analysis and revised the manuscript. Jane Hocking contributed to data analysis and interpretation, and drafting and revising the manuscript. Biangtung Langkham and Nick Crofts contributed to study conceptualisation and design, and drafting and revising the manuscript.
